# Gene Expression Network Analysis of ETV1 Reveals KCTD10 as a Novel Prognostic Biomarker in Gastrointestinal Stromal Tumor

**DOI:** 10.1371/journal.pone.0073896

**Published:** 2013-08-19

**Authors:** Daisuke Kubota, Akihiko Yoshida, Hitoshi Tsuda, Yoshiyuki Suehara, Taketo Okubo, Tsuyoshi Saito, Hajime Orita, Koichi Sato, Takahiro Taguchi, Takashi Yao, Kazuo Kaneko, Hitoshi Katai, Akira Kawai, Tadashi Kondo

**Affiliations:** 1 Division of Pharmacoproteomics, National Cancer Centre Research Institute, Tokyo, Japan; 2 Department of Orthopedic Surgery, Juntendo University School of Medicine, Tokyo, Japan; 3 Pathology and Clinical Laboratory Division, National Cancer Centre Hospital, Tokyo, Japan; 4 Department of Human Pathology, Juntendo University School of Medicine, Tokyo, Japan; 5 Department of Surgery, Juntendo University Shizuoka Hospital, Shizuoka, Tokyo, Japan; 6 Department of Human Health and Medical Science, Graduate School of Kuroshio Science, Kochi University, Kochi, Japan; 7 Department of Surgical Oncology Division, National Cancer Centre Hospital, Tokyo, Japan; 8 Division of Musculoskeletal Oncology, National Cancer Centre Hospital, Tokyo, Japan; University of Hong Kong, Hong Kong

## Abstract

**Background:**

Prognostic biomarkers are required for risk stratification therapy in the patients with gastrointestinal stromal tumor (GIST). In this study, we aimed to identify prognostic biomarkers in GIST. We assessed the prognostic value of E twenty-six variant 1 (ETV1), a recently identified transcription factor unique to GIST. We also examined the clinical utility and functions of its downstream gene, potassium channel tetramerization domain containing protein 10 (KCTD10).

**Methods:**

The levels of ETV1 and KCTD10 were evaluated immunohistochemically in 112 patients with GIST treated at two hospitals. The functional properties of KCTD10 were examined by gene silencing assay in cultured GIST cells.

**Results:**

Immunohistochemistry revealed that ETV1 expression in GIST had no prognostic significance. In contrast, the disease-free survival rate was 88.5% in patients with KCTD10-positive tumors and 55.8% in those with KCTD10-negative tumors (*p* <0.0001). KCTD10 was an independent prognostic factor (*p* <0.05). In the low-risk classification group, KCTD10 was significantly associated with favorable prognosis (*p* = 0.0008). Gene silencing of KCTD10 increased cell proliferation and invasion, suggesting that KCTD10 has a tumor-suppressive function.

**Conclusions:**

The GIST-specific transcription factor ETV1 may have no prognostic potential, whereas its downstream gene KCTD10 is associated with a favorable prognosis. Our study indicated the novel prognostic utility of KCTD10 in GIST, and suggested its tumor-suppressive effects on GIST cells. Further validation studies of KCTD10 for clinical applications, and functional verification of KCTD10 for better understanding of molecular basis of malignant phenotypes are worth challenging in GIST.

## Introduction

Gastrointestinal stromal tumor (GIST) is the most common primary sarcoma of the gastrointestinal tract [1]. The clinical course of GIST ranges from negligible, as in cases of microGIST, to highly malignant and inoperable disease [2–5]. GIST is characterized by the presence of mutations in receptor tyrosine kinases: activating mutations are present in KIT and PDGFRA in approximately 80% and 10% of GISTs, respectively [1]. Treatment with imatinib mesylate (Gleevec; Novartis), a receptor tyrosine kinase inhibitor, is reportedly effective in patients with metastatic GIST [6,7], and adjuvant imatinib treatment prolongs both survival and the time to metastasis [8]. Estimation of the postoperative risk of metastasis becomes more important in the management of operable GIST, because approximately 60% of GIST patients can be cured by surgical resection alone, and imatinib therapy may benefit only a limited number of patients [9]. Previous genetic and epigenetic studies have revealed many prognostic molecular biomarkers (Data S1). Such studies can lead to the discovery of practical molecular biomarkers that reflect the mechanisms responsible for various degrees of risk, or can be considered as independent prognostic parameters.

A recent study has revealed that E twenty-six variant 1 (ETV1), which belongs to a family of transcription factors, is expressed specifically in GIST [10]. In vitro studies have suggested that ETV1 may functionally contribute to cell cycle progression and tumorigenicity. Although clinical applications of ETV1 seem feasible because of its oncogenic role in GIST cells, ETV1 protein is expressed in only 50.4% of GIST cases and therefore its prognostic significance has been controversial [11]. While one gene-silencing assay listed 48 genes that were possibly under the control of ETV1, there has been no evidence to support their clinical value [10]. ETV1 is the only transcription factor specific to GIST that has been reported to date; therefore, evaluation of its clinical applications and downstream genes is warranted in order to obtain a clearer picture of the molecular characteristics of GIST.

Previously, we identified the prognostic significance of KCTD12 (potassium channel tetramerization domain containing protein 12, pfetin) in GIST using a proteomic approach. Immunohistochemical validation studies have demonstrated the prognostic utility of KCTD12 in 486 GIST cases from 6 hospitals [12–16]. KCTD10, another KCTD family gene, has been listed as one of the genes regulated by ETV1 [10]. Therefore, we hypothesized that KCTD family genes may be useful for assessing the malignant potential of GIST cells.

The aim of the present study was to establish novel prognostic biomarkers in GIST. We examined the expression of ETV1 and KCTD10 immunohistochemically in primary GIST tissues, and also evaluated the functional properties of KCTD10 in GIST cells.

## Materials and Methods

### 1: Patients

Our protein expression study using Western blotting included patients with GIST, osteosarcoma, rhabdomyosarcoma, alveolar soft part sarcoma, and epithelioid sarcoma. All were treated at the National Cancer Centre Hospital between 1996 and 2010. The clinicopathological features of the 6 GIST cases examined in this study are listed in Table S1. GIST cases 1-3 did not have metastasis more than 2 years after surgery, and GIST cases 4-6 developed metastasis within one year after surgery. None of the 6 patients received adjuvant treatment with imatinib mesylate. The immunohistochemical study included 112 GIST cases: 40 from the Juntendo University Shizuoka Hospital treated during 1995–2009 and 72 at the Juntendo University Hospital treated during 2000–2009. All the patients underwent surgical resection with curative intent and were not given adjuvant treatment, including imatinib mesylate. Diagnosis of GIST was based on the WHO classification system for soft-tissue tumors [17]. Overexpression of c-kit in tumor cells was confirmed in all 112 GIST cases by immunohistochemistry (CD117 antibody; DAKO Japan Corp., Tokyo, Japan). Risk classification was performed according to the established risk classification system [18]. The clinicopathological features of the 112 GIST patients are listed in Table S2 and summarized in Table 1. In our previous study, we examined the expression level of KCTD12 in the same 112 GIST patients, and the results were summarized in Table 2 and Table S2 [14,15].

**Table 1:** Summary of clinical and pathological characteristics of the GIST cases used for immunohistochemistry.

Variable		Number of cases
Age	<60	40 (35.7%)
	≥60	72 (64.3%)
Sex	Female	65 (58.0%)
	Male	47 (42.0%)
Site	Stomach	85 (75.9%)
	Non-gastric	27 (24.1%)
Histology	Spindle	99 (88.4%)
	Epithelioid	10 (8.9%)
	Mixed	3 (2.7%)
Size (cm)	<5	64 (57.1%)
	5–15	44 (39.3%)
	≥15	4 (3.6%)
Necrosis	Present	24 (21.4%)
	Absent	88 (78.6%)
Miettinen’s Risk classification	Low	73 (65.2%)
	Intermediate	12 (10.7%)
	High	27 (24.1%)
Post-operative metastasis	Present	23 (20.5%)
	Absent	89 (79.5%)

**Table 2:** Summary of uni- and multi-variate analysis.

								Disease-free survival		Multivariate analysis of disease-free survival by Cox regression		
Variable	Number of cases	ETV1 positive	ETV1 negative	Correlation (ETV1) χ^2^ *P* value	KCTD10 positive	KCTD10 negative	Correlation (KCTD10) χ^2^ *P* value	Rate (%)	Log-rank (*P* value)	*P* value	Relative risk	95% confidence interval
Age												
<60	40	25	15	0.666	30	10	0.383	75	0.3141			
≥60	72	42	30		59	13		81.94				
Sex												
Female	45	25	20	0.45	34	11	0.401	82.22	0.709			
Male	67	42	25		55	12		77361				
Site												
Stomach	85	46	39	0.015	68	17	0.535	80				
Small intestine	22	19	3		18	4		77.27	0.7584			
Other	5	2	3		3	2		80				
Histology												
Spindle	99	58	41	0.759	77	22	0.443	79.8				
Epithelioid	10	7	3		9	1		80	0.9048			
Mixed	3	2	1		3	0		66.67				
Size (cm)												
<5	64	43	21	0.019	51	13	0.02	92.19	0.0001	0.118	0.31	0.071-1.349
5–15	44	24	20		37	7		68.18				
≥15	4	0	4		1	3		0				
Necrosis												
Present	24	15	9	0.763	22	2	0.095	79.17	0.7289			
Absent	88	52	36		67	21		79.55				
Risk classification^a^												
Low	73	46	27	0.028	60	13	0.619	94.52	<0.0001	< 0.0001	6.896	2.532-18.405
Intermediate	12	10	2		9	3		75				
High	27	11	16		20	7		40.74				
Post-operative metastasis												
Present	23	57	32	0.073	12	11	<0.0001					
Absent	89	10	13		77	12						
ETV1							0.401					
Positive	67	67	0	-	55	12		85.07	0.2667			
Negative	45	0	45		34	11		71.11				
KCTD10												
Positive	89	55	34	0.401	89	0	-	86.52	<0.0001	< 0.0001	0.125	0.042-0.368
Negative	23	12	11		0	23		52.17				
^a^ Risk classification based on tumor size, location, and mitotic rate (Miettinen’s classification, ref. 16).												

### Ethics Statement

Written informed consent was obtained from all patients, and this study was approved by the ethics committees of Juntendo University and the National Cancer Centre.

### 2: Immunological Examination

The expression levels of ETV1 and KCTD10 were examined in the identical sample set by Western blotting. The proteins were extracted from frozen samples of primary tumors, and subjected to SDS-PAGE. The separated proteins were transferred to a membrane, and reacted with antibody against ETV1 (ab81086, 1:200 dilution; Abcam, Cambridge, UK) or KCTD10 (HPA014273, 1:1000 dilution; Sigma-Aldrich, Saint Louis, MO). After the membrane had been treated with the secondary antibody (GE Healthcare Biosceinces), the immunocomplex was detected by ECL prime (GE Healthcare Biosciences) and LAS-3000 (FUJIFILM, Tokyo, Japan).

The expression levels of both ETV1 and KCTD10 were immunohistochemically examined in the identical 112 GIST patients as described in our previous reports [12–15]. Briefly, 4-µm-thick formalin-fixed, paraffin-embedded tissue sections were autoclaved in 10 mmol/L citrate buffer (pH 6.0) at 121°C for 30 min and incubated with anti-ETV1 (ab81086, 1:200 dilution; Abcam) and anti-KCTD10 (HPA014273, 1:150 dilution; Sigma-Aldrich, Saint Louis, MO) antibodies. Immunostaining was carried out by the streptavidin–biotin peroxidase method using an ABC complex/horseradish peroxidase kit (DAKO). One pathologist (A.Y.) and one clinician (D.K.) reviewed the stained sections blinded to the clinical data (age, gender, anatomic site, metastasis, and clinical endpoints such as time to metastasis and survival period). In our previous studies, we had considered that cases in which >20% of tumor cells were stained with the antibody against KCTD12 were KCTD12-positive, whereas those in which <20% of tumor cells were stained with the antibody against KCTD12 were KCTD12-negative [12–15]. In this study, we employed the same criteria for positive and negative expression of ETV1 and KCTD10.

### 3: KCTD10 Functional Assay

The human GIST T1 cells were cultured in Dulbecco’s modified Eagle medium supplemented with 10% fetal bovine serum (Life Technologies, Carlsbad, California) [19]. KCTD10-specific siRNAs were purchased from Sigma-Aldrich (HS01-00108591, HS01-00108592, and HS01-00108593, St. Louis, MO), and control stealth siRNA was from Life Technologies. A total of 5 × 10^3^ cells were seeded into each well of a 96-well plate (Coaster, Cambridge, MA). The following day, the cell monolayer was washed with pre-warmed sterile phosphate-buffered saline. Cells were transfected with the appropriate siRNA using DharmaFECT transfection reagents (Thermo, Fisher, Waltham, MA) in accordance with the manufacturer’s protocol. Twenty-four hours later, the cultured medium of the transfected cells was switched to medium A, whereas the conditioned medium was not changed. Cell proliferation was examined every 3 days after transfection by the tetrazolium-based colorimetric MTT assay, 20 µl of reagent from the Cell Counting Kit-8 (Dojindo, Kumamoto, Japan) being added to each well. After 2 h of incubation at 37 °C, the optical density was measured at a wavelength of 450 nm using a microplate reader.

Cell invasion was evaluated using the BD BioCoat^TM^ Invasion Chamber (BD Bioscience, Franklin Lakes, NJ) in accordance with the manufacturer’s protocol. Briefly, cells were transfected with the appropriate siRNA using DharmaFECT transfection reagents for 24 h. Cells were seeded onto the membrane of the upper chamber of the transwell at a concentration of 5 × 10^5^ in 500µl of serum-free medium. The medium in the lower chamber contained 10% fetal calf serum as a source of chemoattractants. Cells that passed through the Matrigel-coated membrane were stained with Diff-Quick (Sysmex, Kobe, Japan) and photographed.

### 4: Statistical Analysis

All statistical analyses were carried out using the χ^2^ test or Fisher’s exact test in cross tables to assess the relationships between expression levels of ETV1 or KCTD10 and clinicopathological factors [20,21]. The primary endpoint of this study was disease-free survival (DFS), calculated as the period from initial resection of the primary tumor to the first evidence of metastasis. All time–event endpoints were computed by the Kaplan–Meier method [22]. Patients who died due to factors unrelated to GIST were excluded at the time of death. Potential prognostic factors were identified by univariate analysis using the log-rank test. Independent prognostic factors were evaluated using the Cox proportional hazards regression model using variables found to be significant at the univariate level (*p* <0.05) [23]. Calculations were carried out using the SPSS statistical software package (IBM, Armonk, NY).

## Results

### 1: Expression of ETV1 does not correlate with poor prognosis in GIST

**Figure 1:**
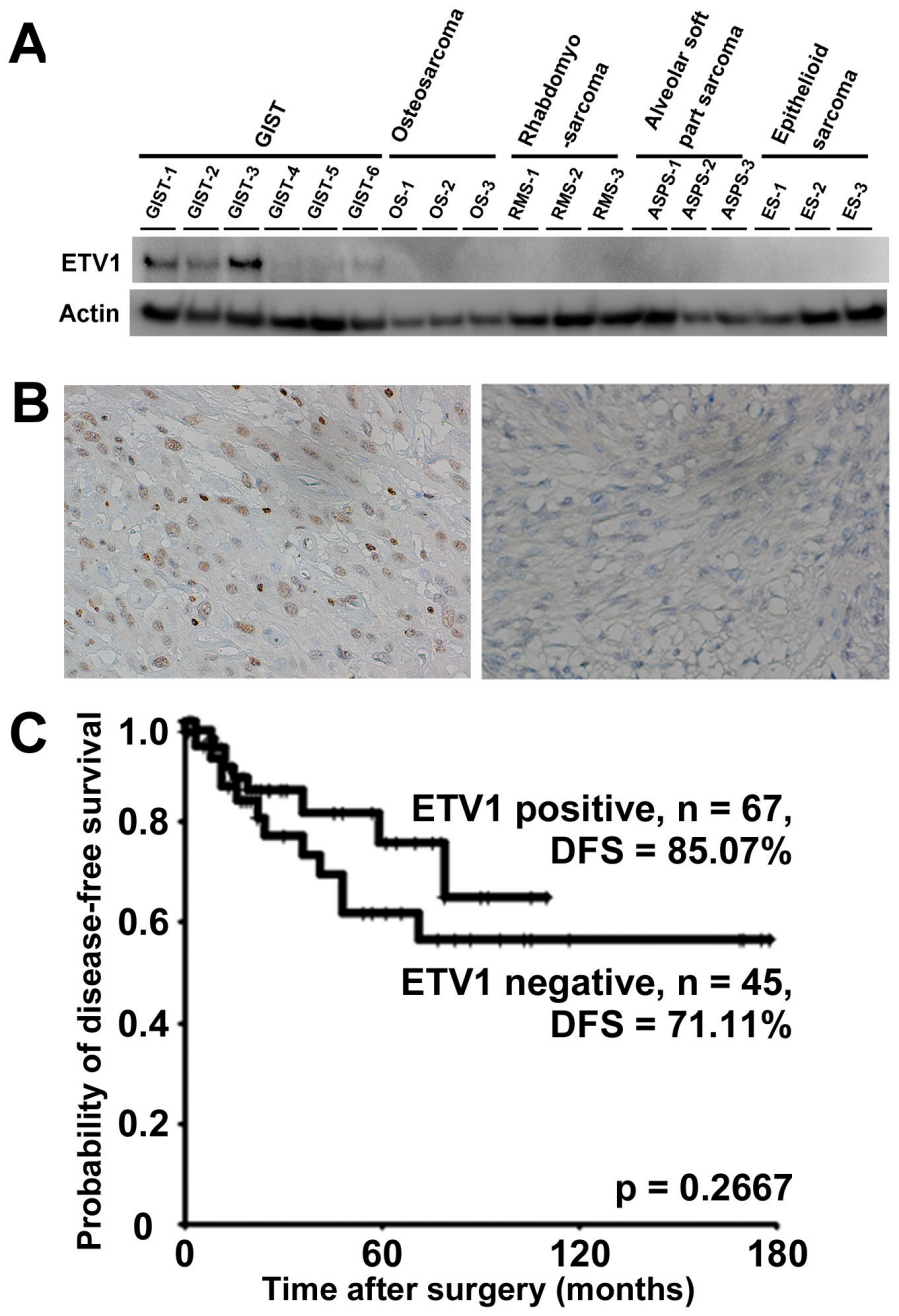
ETV1 expression in GIST tissues evaluated by Western blotting (A). Expression of ETV1 was observed only in GIST cases, especially those with a favorable outcome (GIST cases 1-3). Specimen of GIST showing strong, uniform nuclear expression of ETV1 (left panel, B) and a case showing negative ETV1 expression (right panel, B). DFS curves for 112 GIST cases (C). No statistically significant differences in DFS were observed between ETV1-positive and -negative GIST cases.

Unique expression of ETV1 in GIST tissues was first confirmed by Western blotting (Figure 1A). The expression level of ETV1 in the primary tumor tissues from GIST cases 1-3, in which metastasis did not develop for more than 2 years after surgery, was clearly higher than in GIST cases 4-6, in which metastasis developed within one year after surgery. No expression of ETV1 was observed in the tissues of other sarcomas.

The prognostic significance of ETV1 was further examined in 112 additional GIST cases. Immunohistochemistry revealed that ETV1 was diffusely localized in the nuclei of the tumor cells (Figure 1B left panel). One case that was immunohistochemically negative for ETV1 was found (Figure 1B right panel). Among the 112 cases examined, 67 (59.8%) were considered to be positive for ETV1. ETV1 expression was significantly correlated with the primary tumor location tumor size, and Miettinen’s risk classification (*p* <0.05, Fisher’s exact test, Table 2). The DFS rate was 85.1% and 71.1% for ETV1-positive and -negative patients, respectively (*p* = 0.267; log-rank test, Figure 1C).

### 2: Expression of KCTD10 is correlated with the prognosis of GIST

**Figure 2:**
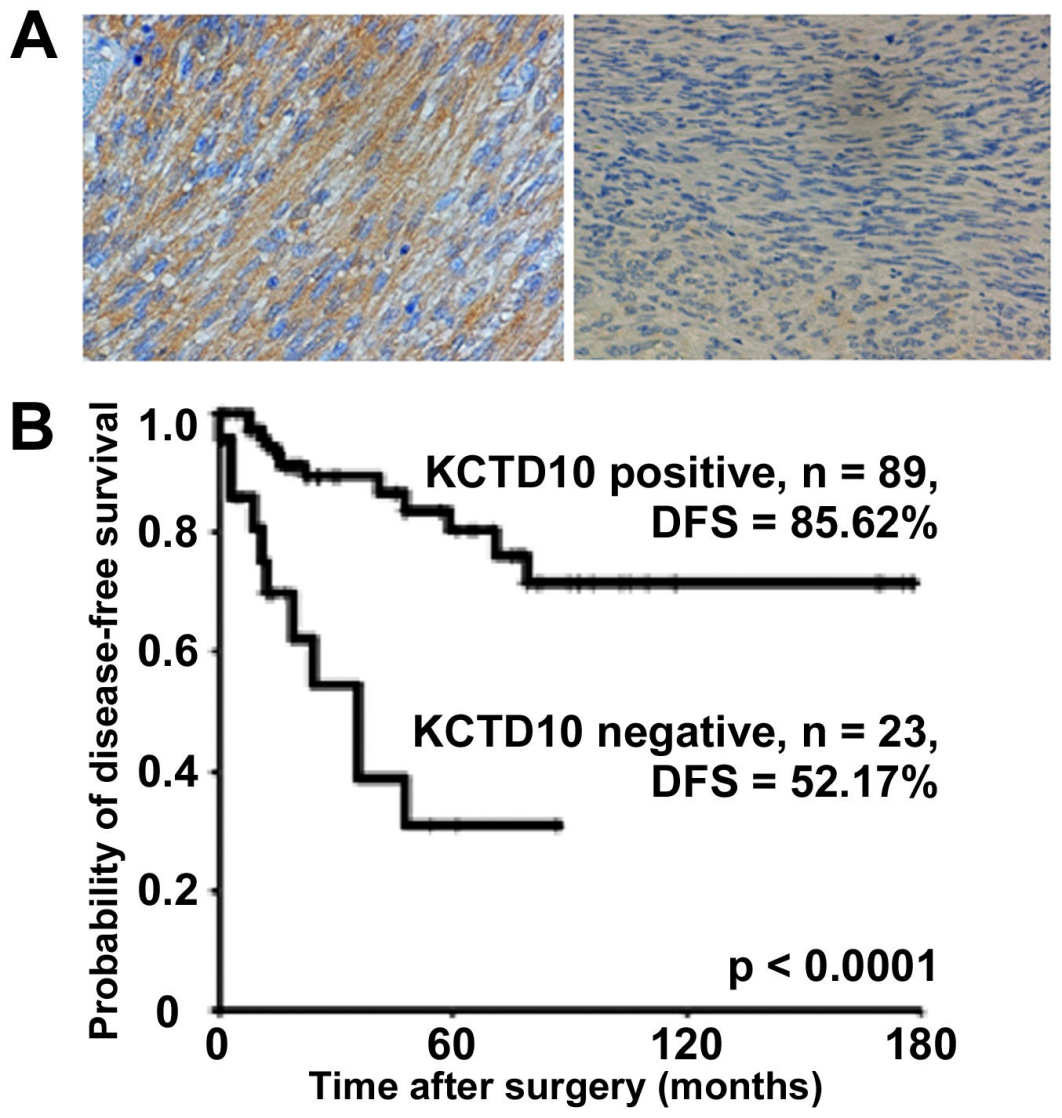
KCTD10 expression and its prognostic significance. Specimen from a GIST patient with no metastasis, showing strong expression of KCTD10 (left panel, B). Lack of KCTD10 expression in a specimen from a GIST patient who developed postoperative metastasis at 19 months after surgery (right panel, B). DFS curves estimated by the Kaplan–Meier method for 112 cases (C). Statistically significant differences in DFS were observed between KCTD10-positive and -negative cases.

A previous study had reported 48 genes that are regulated by ETV1, including KCTD10; downregulation of ETV1 by shRNA inhibited KCTD10 expression, suggesting that KCTD10 expression might be attributable to ETV1-induced malignant phenotypes of GSIT cells [10]. Therefore, we examined the prognostic utility of KCTD10. We examined the expression level of KCTD10 in the identical sample set that we examined for ETV1 expression (Figure S1). We found that the expression level of KCTD10 was higher in the patients with favorable prognosis than those with poor prognosis with statistical significances (*p* = 0.031). The expression of KCTD10 was observed in the primary tumors of osteosarcoma, rhabdomyo sarcoma, alveolar soft part sarcoma, and epithelioid sarcoma with various levels (Figure S1). Immunohistochemistry showed that KCTD10 was highly expressed in sample S-1, obtained from a patient with no postoperative metastasis (Figure 2A left panel; Table S1). KCTD10 was diffusely expressed in the membrane and cytoplasm of the tumor cells (Figure 2A left panel), while no KCTD10 expression was observed in sample S-7 from a patient who developed postoperative metastasis at 19 months (Figure 2A right panel; Table S1). KCTD10 expression was significantly correlated with tumor size and postoperative metastasis (*p* <0.05, Fisher’s exact test, Table 2). Kaplan–Meier survival analysis revealed that KCTD10 expression in GIST was significantly correlated with the DFS rate, which was 85.62% and 52.17% for KCTD10-positive and -negative cases, respectively (*p* <0.0001; log-rank test, Figure 2B). Multivariate analysis revealed that, among other clinicopathological parameters of GIST, the risk classification and KCTD10 were independent prognostic factors (*p* = 0.004, Table 2). These observations suggest that the expression level of KCTD10 in GIST is associated with a favorable prognosis.

### 3: Survival analysis of KCTD10 expression in relation to risk classification

**Figure 3:**
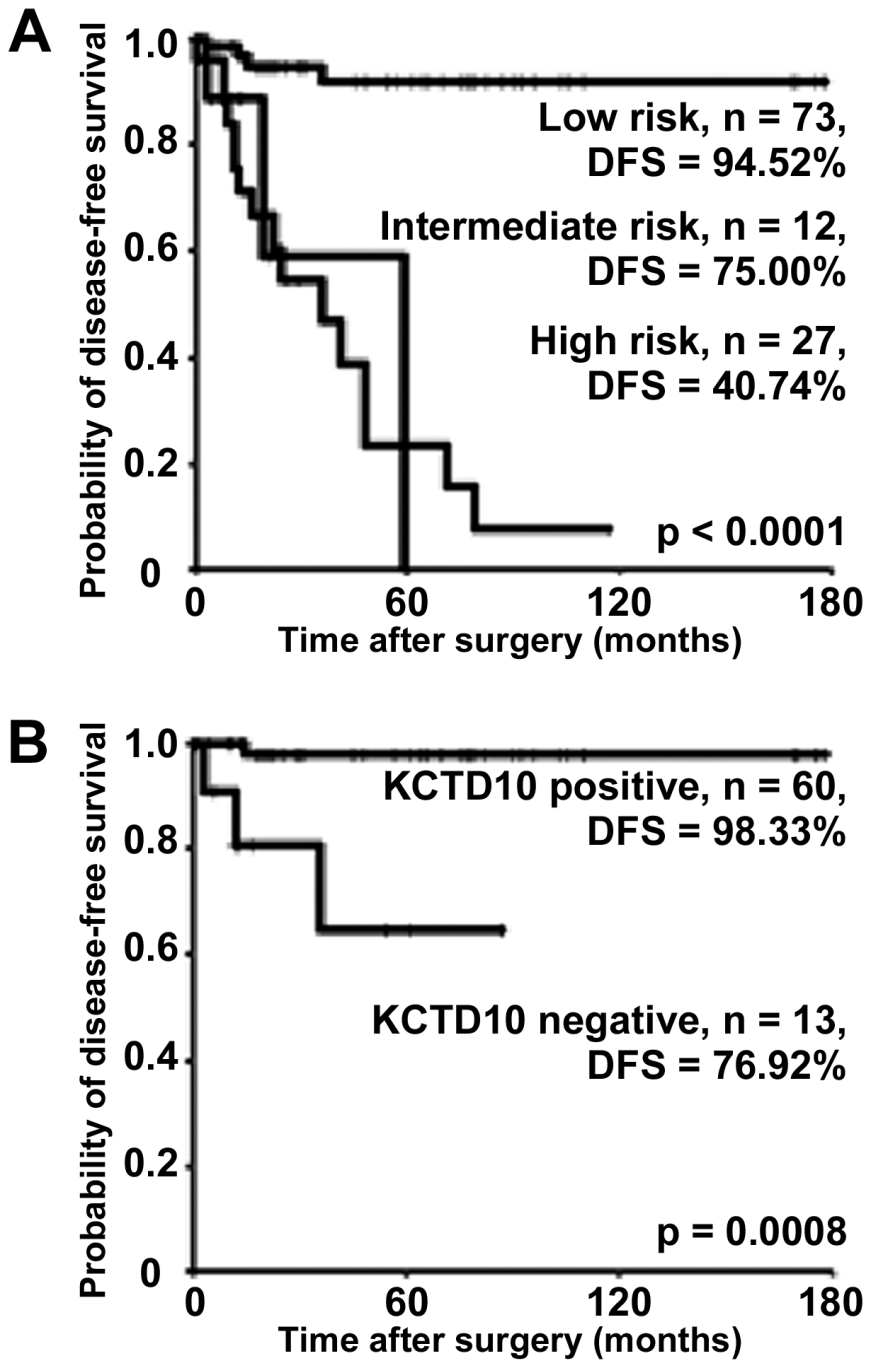
Prognostic potential of KCTD10 according to Miettinen’s risk classification. Kaplan–Meier curves for DFS in all 112 cases based on the risk classification (A). Prognostic significance of KCTD10 was demonstrated in the low-risk group (B).

We investigated the relationship between Miettinen’s risk classification and DFS in the 112 GIST cases. On the basis of the risk classification, metastasis was observed in 4 of 73 patients (5.5%) in the low-risk group, 3 of 12 patients (25.0%) in the intermediate-risk group, and 16 of 27 patients (59.3%) in the high-risk group (Figure 3A). Among low-risk patients, those with a KCTD10-negative primary tumor had a lower DFS rate (*p* = 0.0008; log-rank test, Figure 3B). No significant differences were evident between intermediate- and high-risk patients, probably because of the small number of cases in these groups (n = 12 and n = 27, respectively). These observations suggest the potential utility of KCTD10 as a prognostic biomarker, especially in low-risk patients.

### 4: In vitro function study of KCTD10

**Figure 4:**
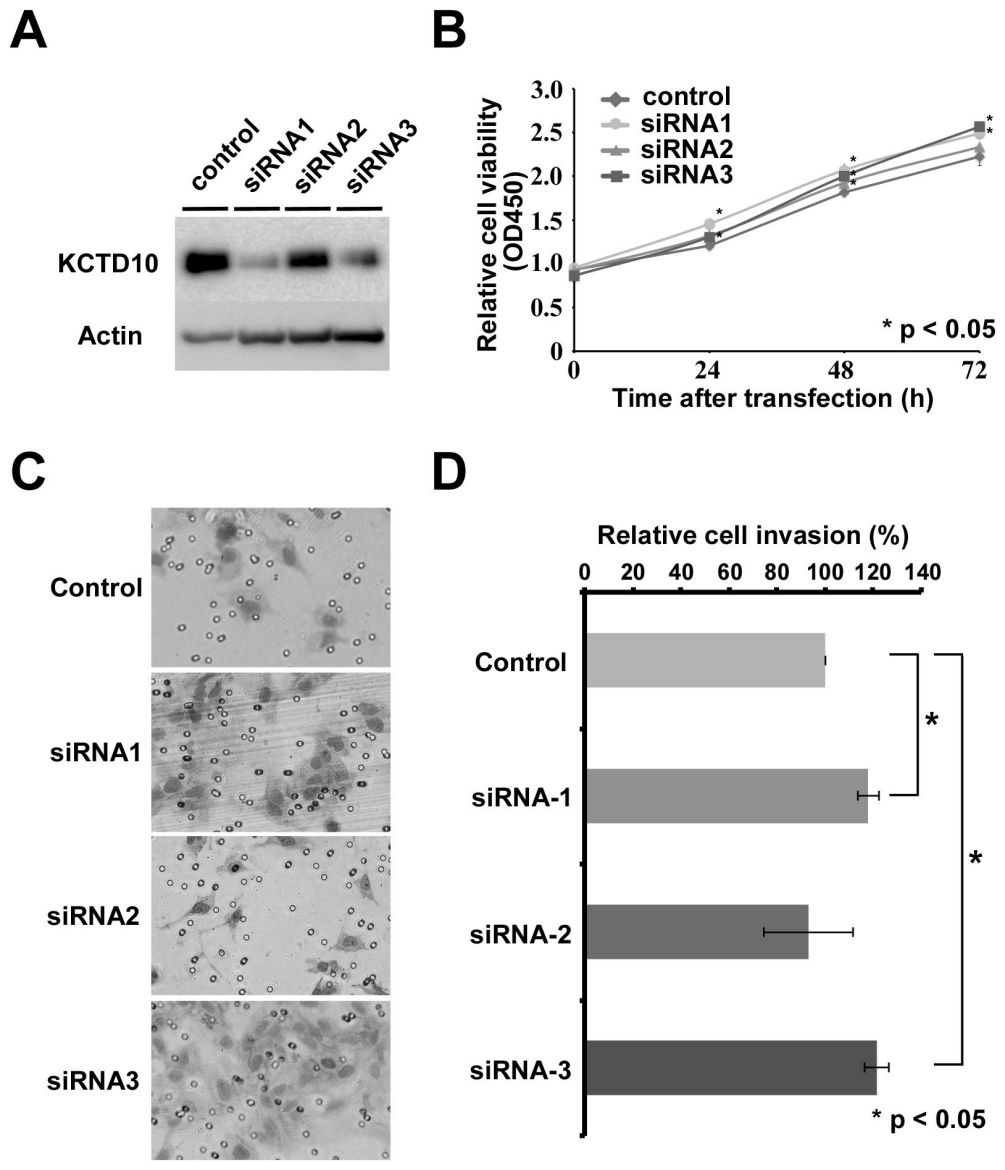
Silencing of KCTD10 by siRNAs and its effects on cell growth and invasion. KCTD10 expression was suppressed by treatment with specific siRNAs against KCTD10 and compared with the control (A). Treatments with siRNAs 1 or 3 decreased the expression of KCTD10 in GIST T1 cells, and proliferation of the cells with decreased KCTD10 expression was significantly inhibited (B). The appearance of invading cells treated with siRNAs (C). The number of invasive cells was significantly (p <0.01) decreased by treatment with siRNAs 1 or 3 (D).

To evaluate the functional roles of KCTD10 in GIST cells, we examined the effects of KCTD10 expression on cell proliferation and invasion (Figure 4). Transfection of GIST T1 cells with siRNAs 1 and 3 against KCTD10 resulted in a remarkable reduction of KCTD10 expression in comparison with the control cells transfected with negative siRNA (Figure 4A). Cell viability assays revealed that transfection of siRNAs 1 and 3 resulted in an increase of cell growth relative to negative control GIST T1 cells, and siRNA2 transfection did not significantly decrease cell proliferation (Figure 4B). In addition, siRNA-mediated silencing of KCTD10 significantly increased the invasive ability of GIST T1 cells in the siRNA1 and siRNA3 groups (p = 0.0143 and p = 0.0134, respectively, Figure 4C and D), but not in the siRNA2 group. These observations suggested that KCTD10 may have tumor-suppressive roles in GIST cells.

## Discussion

In GISTs, adjuvant therapy with imatinib has prolonged the period until development of postoperative metastasis, and patients have exhibited longer survival times [8]. However, approximately 60% of GIST patients can be cured by surgical resection alone, and imatinib treatment may benefit only a limited number of individuals [9]. Therefore, there is an urgent need for novel biomarkers that would allow assessment of the malignant potential of GIST cells, prognostication, and evaluation of possible therapies. In the present study, we immunohistochemically examined the prognostic value of ETV1 protein and its downstream protein, KCTD10, in 112 cases of GIST. ETV1 is a novel transcription factor that shows specific expression in GISTs, and is essential for the growth of GIST cells [10]. The ETV1 transcriptional program is regulated by activated KIT, which prolongs ETV1 protein stability and cooperates with ETV1 to promote tumorigenesis. These observations suggest that ETV1 and its downstream genes may be candidate prognostic biomarkers for GIST. In contrast, Birner et al. reported that expression of ETV1 was observed only half of the GIST cases they examined, and that ETV1 expression was of no prognostic significance in GIST [11]. Therefore, further investigation of ETV1 in GISTs has been warranted.

First, using Western blotting, we confirmed the unique expression of ETV1 in the GIST cases we examined, especially those with a favorable outcome (Figure 1A). This prompted us to investigate the correlation between ETV1 expression and outcome after surgery in additional cases. Our immunohistochemical analysis revealed ETV1 expression in only 67 of 112 GIST cases (59.8%) (Table 2). Although the patients with ETV1-positive primary tumors tended to have a favorable outcome, there was no significant correlation between ETV1 expression and DFS (Figure 1C). Therefore we concluded that ETV1 had no prognostic utility. These observations were consistent with a previous immunohistochemical study in which the rate of pfetin positivity was 50.4%, and no evident prognostic utility of ETV1 was detected [11].

A previous report has indicated that KCTD10 is under the control of ETV1 [10], and using a proteomic approach we have identified KCTD12 as a novel prognostic biomarker [12]. Here, we found that KCTD10 had novel prognostic utility in the identical GIST cases that we examined ETV1 expression; patients with KCTD10-positive primary tumors had a better outcome than those with KCTD10-negative primary tumors (Figure 4). It was noteworthy that the expression level of KCTD10 was able to predict metastasis in low-risk patients (Figure 4B). About 50% of GISTs are small, asymptomatic tumors that are mostly diagnosed accidentally, and these are considered to have a low risk of relapse. However, a small number of low-risk patients do develop postoperative metastasis, and therefore a prognostic biomarker for such patients is urgently required [2,3]. KCTD10 may be useful for this purpose. In the present study, no significant differences in KCTD10 expression were evident between the intermediate- and high-risk groups, and therefore further evaluation of the prognostic value of KCTD10 in a large-scale validation study would seem warranted. We previously examined the prognostic utility of KCTD12 [14,15], and KCTD12 was an independent prognostic factor in this study (Table 2). The expression level of both KCTDs10 and 12 were parallel with favorable prognosis. Presently it is hard to decide which KCTD will be more practical in clinical settings, and the further validation study should be performed for both KCTD10 and 12.

We revealed that KCTD10 may exert tumor-suppressive effects in GIST. The functions of KCTD10 are still unclear. KCTD10 belongs to the polymerase delta-interacting protein 1 family, which is induced by tumor necrosis factor alpha and IL-6, and plays a role in DNA synthesis [24]. A previous study has shown that KCTD10 interacts with proliferating cell nuclear antigen, and increases DNA synthesis and proliferation of lung cancer cells [25]. Expression of the KCTD10 transcript was reduced when ETV1 was silenced in GIST cells, suggesting that KCTD10 may be responsible for the malignant features elicited by ETV1 [10]. In contrast, the results of our immunohistochemical study were contrary to expectation: the expression level of KCTD10 was higher in patients with a favorable outcome (Figure 4, Table 2). Recently, Hu et al. reported that KCTD10 inhibited the transcriptional activities of NF-κB and AP-1 [26]. These inhibitory effects were strongly promoted by treatment with a proteasome inhibitor, as KCTD10 expression is regulated through ubiquitination and degradation [26]. Therefore, further investigation of KCTD10, especially in vivo experiments, would be of interest from the view point of biomarker research and novel therapeutic intervention.

In conclusion, we anticipate that our present data for KCTD10 will lead to the clarification of a novel immunohistochemical biomarker appropriate for evaluation of metastasis risk in patients with GIST. Prognostication using KCTD10 may help to optimize the treatment strategy for patients with GIST.

## Supporting Information

Data S1 **Reported molecular biomarkers in GIST.**
(DOCX)

Figure S1 **KCTD10 expression in GIST tissues evaluated by Western blotting (A).** Expression of KCTD10 was broadly observed in sarcoma cases. Statistically significant differences in the expression level were observed between the patient groups with different prognosis (B).
(JPG)

Table S1 **Clinical and pathological characteristics of the GIST cases used for Western blotting.**
(XLSX)

Table S2 **Clinical and pathological characteristics of the GIST cases used for immunohistochemistry.**
(XLSX)
