# Supplementary material for: Gene Expression Network Analysis of ETV1 Reveals KCTD10 as a Novel Prognostic Biomarker in Gastrointestinal Stromal Tumor
Source: PLoS One. 2013 Aug 19;8(8):e73896. doi: 10.1371/journal.pone.0073896 (PMC3747077; doi:10.1371/journal.pone.0073896)
Supplement: Data S1 — Reported molecular biomarkers in GIST. (DOCX) [file pone.0073896.s001.docx]

Supplementary data

Reported molecular biomarkers in GIST [^1-41^](#_ENREF_1)

1. Edris B, Espinosa I, Muhlenberg T, Mikels A, Lee CH, Steigen SE, Zhu S, Montgomery KD, Lazar AJ, Lev D, Fletcher JA, Beck AH, et al. ROR2 is a novel prognostic biomarker and a potential therapeutic target in leiomyosarcoma and gastrointestinal stromal tumour. J Pathol 2012;227:223-33.

2. Niinuma T, Suzuki H, Nojima M, Nosho K, Yamamoto H, Takamaru H, Yamamoto E, Maruyama R, Nobuoka T, Miyazaki Y, Nishida T, Bamba T, et al. Upregulation of miR-196a and HOTAIR drive malignant character in gastrointestinal stromal tumors. Cancer Res 2012;72:1126-36.

3. Fujita A, Yamamoto H, Imamura M, Nakamura N, Maehara Y, Tsuneyoshi M, Oda Y. Expression level of the mitotic checkpoint protein and G2-M cell cycle regulators and prognosis in gastrointestinal stromal tumors in the stomach. Virchows Arch 2012;460:163-9.

4. Okamoto Y, Sawaki A, Ito S, Nishida T, Takahashi T, Toyota M, Suzuki H, Shinomura Y, Takeuchi I, Shinjo K, An B, Ito H, et al. Aberrant DNA methylation associated with aggressiveness of gastrointestinal stromal tumour. Gut 2012;61:392-401.

5. Zander H, Rawnaq T, von Wedemeyer M, Tachezy M, Kunkel M, Wolters G, Bockhorn M, Schachner M, Izbicki JR, Kaifi J. Circulating levels of cell adhesion molecule L1 as a prognostic marker in gastrointestinal stromal tumor patients. BMC Cancer 2011;11:189:1-7.

6. Wozniak A, Rutkowski P, Piskorz A, Ciwoniuk M, Osuch C, Bylina E, Sygut J, Chosia M, Rys J, Urbanczyk K, Kruszewski W, Sowa P, et al. Prognostic value of KIT/PDGFRA mutations in gastrointestinal stromal tumours (GIST): Polish Clinical GIST Registry experience. Ann Oncol 2012;23:353-60.

7. Setoguchi T, Kikuchi H, Yamamoto M, Baba M, Ohta M, Kamiya K, Tanaka T, Baba S, Goto-Inoue N, Setou M, Sasaki T, Mori H, et al. Microarray analysis identifies versican and CD9 as potent prognostic markers in gastric gastrointestinal stromal tumors. Cancer Sci 2011;102:883-9.

8. Ruiz-Tovar J, Diez-Tabernilla M, Housari G, Martinez-Molina E, Sanjuanbenito A. Gastrointestinal stromal tumors: actin expression, a new prognostic factor? Am Surg 2010;76:1244-50.

9. Arne G, Kristiansson E, Nerman O, Kindblom LG, Ahlman H, Nilsson B, Nilsson O. Expression profiling of GIST: CD133 is associated with KIT exon 11 mutations, gastric location and poor prognosis. Int J Cancer 2011;129:1149-61.

10. Braggio E, Braggio Dde A, Small IA, Lopes LF, Valadao M, Gouveia ME, Moreira Ados S, Linhares E, Romano S, Bacchi CE, Renault IZ, Guimaraes DP, et al. Prognostic relevance of KIT and PDGFRA mutations in gastrointestinal stromal tumors. Anticancer Res 2010;30:2407-14.

11. Rawnaq T, Kunkel M, Bachmann K, Simon R, Zander H, Brandl S, Sauter G, Izbicki JR, Kaifi JT. Serum midkine correlates with tumor progression and imatinib response in gastrointestinal stromal tumors. Ann Surg Oncol 2011;18:559-65.

12. Hsu KH, Tsai HW, Lin PW, Hsu YS, Shan YS, Lu PJ. Osteopontin expression is an independent adverse prognostic factor in resectable gastrointestinal stromal tumor and its interaction with CD44 promotes tumor proliferation. Ann Surg Oncol 2010;17:3043-52.

13. Hsu KH, Tsai HW, Lin PW, Hsu YS, Shan YS, Lu PJ. Clinical implication and mitotic effect of CD44 cleavage in relation to osteopontin/CD44 interaction and dysregulated cell cycle protein in gastrointestinal stromal tumor. Ann Surg Oncol 2010;17:2199-212.

14. Silva M, Veiga I, Ribeiro FR, Vieira J, Pinto C, Pinheiro M, Mesquita B, Santos C, Soares M, Dinis J, Santos L, Lopes P, et al. Chromosome copy number changes carry prognostic information independent of KIT/PDGFRA point mutations in gastrointestinal stromal tumors. BMC Med 2010;8:26.

15. Martinho O, Gouveia A, Silva P, Pimenta A, Reis RM, Lopes JM. Loss of RKIP expression is associated with poor survival in GISTs. Virchows Arch 2009;455:277-84.

16. Rawnaq T, Schwarzenbach H, Schurr PG, Freise K, Brandl S, Izbicki JR, Kaifi JT. Monitoring of loss of heterozygosity in serum microsatellite DNA among patients with gastrointestinal stromal tumors indicates tumor recurrence. J Surg Res 2011;169:31-5.

17. Wei YC, Li CF, Yu SC, Chou FF, Fang FM, Eng HL, Uen YH, Tian YF, Wu JM, Li SH, Huang WW, Li WM, et al. Ezrin overexpression in gastrointestinal stromal tumors: an independent adverse prognosticator associated with the non-gastric location. Mod Pathol 2009;22:1351-60.

18. Song Z, Wang JL, Pan YL, Tao DY, Gan MF, Huang KE. Survival and prognostic factors analysis in surgically resected gastrointestinal stromal tumor patients. Hepatogastroenterology 2009;56:149-53.

19. Hou YY, Grabellus F, Weber F, Zhou Y, Tan YS, Li J, Shen KT, Qin J, Sun YH, Qin XY, Bockhorn M, Gerken G, et al. Impact of KIT and PDGFRA gene mutations on prognosis of patients with gastrointestinal stromal tumors after complete primary tumor resection. J Gastrointest Surg 2009;13:1583-92.

20. Yamaguchi U, Nakayama R, Honda K, Ichikawa H, Hasegawa T, Shitashige M, Ono M, Shoji A, Sakuma T, Kuwabara H, Shimada Y, Sasako M, et al. Distinct gene expression-defined classes of gastrointestinal stromal tumor. J Clin Oncol 2008;26:4100-8.

21. Perez D, Herrmann T, Jungbluth AA, Samartzis P, Spagnoli G, Demartines N, Clavien PA, Marino S, Seifert B, Jaeger D. Cancer testis antigen expression in gastrointestinal stromal tumors: new markers for early recurrence. Int J Cancer 2008;123:1551-5.

22. Mearadji A, den Bakker MA, van Geel AN, Eggermont AM, Sleijfer S, Verweij J, de Wilt JH, Verhoef C. Decrease of CD117 expression as possible prognostic marker for recurrence in the resected specimen after imatinib treatment in patients with initially unresectable gastrointestinal stromal tumors: a clinicopathological analysis. Anticancer Drugs 2008;19:607-12.

23. Braconi C, Bracci R, Bearzi I, Bianchi F, Costagliola A, Catalani R, Mandolesi A, Ranaldi R, Galizia E, Cascinu S, Rossi G, Giustini L, et al. KIT and PDGFRalpha mutations in 104 patients with gastrointestinal stromal tumors (GISTs): a population-based study. Ann Oncol 2008;19:706-10.

24. Perez D, Demartines N, Meier K, Clavien PA, Jungbluth A, Jaeger D. Protein S100 as prognostic marker for gastrointestinal stromal tumors: a clinicopathological risk factor analysis. J Invest Surg 2007;20:181-6.

25. Takeyama H, Funahashi H, Sawai H, Takahashi H, Yamamotorm M, Akamo Y, Manabe T. Expression of alpha6 integrin subunit is associated with malignancy in gastric gastrointestinal stromal tumors. Med Sci Monit 2007;13:CR51-6.

26. Kaifi JT, Fiegel HC, Rafnsdottir SL, Aridome K, Schurr PG, Reichelt U, Wachowiak R, Kleinhans H, Yekebas EF, Mann O, Ichihara-Tanaka K, Muramatsu T, et al. Midkine as a prognostic marker for gastrointestinal stromal tumors. J Cancer Res Clin Oncol 2007;133:431-5.

27. Huang HY, Huang WW, Lin CN, Eng HL, Li SH, Li CF, Lu D, Yu SC, Hsiung CY. Immunohistochemical expression of p16INK4A, Ki-67, and Mcm2 proteins in gastrointestinal stromal tumors: prognostic implications and correlations with risk stratification of NIH consensus criteria. Ann Surg Oncol 2006;13:1633-44.

28. Kaifi JT, Wagner M, Schurr PG, Wachowiak R, Reichelt U, Yekebas EF, Mann O, Vashist Y, Kleinhans H, Link BC, Strate T, Kalinin V, et al. Allelic loss of Hox11L1 gene locus predicts outcome of gastrointestinal stromal tumors. Oncol Rep 2006;16:915-9.

29. Schurr P, Wolter S, Kaifi J, Reichelt U, Kleinhans H, Wachowiak R, Yekebas E, Strate T, Kalinin V, Simon R, Sauter G, Schaefer H, et al. Microsatellite DNA alterations of gastrointestinal stromal tumors are predictive for outcome. Clin Cancer Res 2006;12:5151-7.

30. Andersson J, Bumming P, Meis-Kindblom JM, Sihto H, Nupponen N, Joensuu H, Oden A, Gustavsson B, Kindblom LG, Nilsson B. Gastrointestinal stromal tumors with KIT exon 11 deletions are associated with poor prognosis. Gastroenterology 2006;130:1573-81.

31. Cho S, Kitadai Y, Yoshida S, Tanaka S, Yoshihara M, Yoshida K, Chayama K. Deletion of the KIT gene is associated with liver metastasis and poor prognosis in patients with gastrointestinal stromal tumor in the stomach. Int J Oncol 2006;28:1361-7.

32. Steinert DM, Oyarzo M, Wang X, Choi H, Thall PF, Medeiros LJ, Raymond AK, Benjamin RS, Zhang W, Trent JC. Expression of Bcl-2 in gastrointestinal stromal tumors: correlation with progression-free survival in 81 patients treated with imatinib mesylate. Cancer 2006;106:1617-23.

33. Haller F, Gunawan B, von Heydebreck A, Schwager S, Schulten HJ, Wolf-Salgo J, Langer C, Ramadori G, Sultmann H, Fuzesi L. Prognostic role of E2F1 and members of the CDKN2A network in gastrointestinal stromal tumors. Clin Cancer Res 2005;11:6589-97.

34. Martin J, Poveda A, Llombart-Bosch A, Ramos R, Lopez-Guerrero JA, Garcia del Muro J, Maurel J, Calabuig S, Gutierrez A, Gonzalez de Sande JL, Martinez J, De Juan A, et al. Deletions affecting codons 557-558 of the c-KIT gene indicate a poor prognosis in patients with completely resected gastrointestinal stromal tumors: a study by the Spanish Group for Sarcoma Research (GEIS). J Clin Oncol 2005;23:6190-8.

35. Nakamura N, Yamamoto H, Yao T, Oda Y, Nishiyama K, Imamura M, Yamada T, Nawata H, Tsuneyoshi M. Prognostic significance of expressions of cell-cycle regulatory proteins in gastrointestinal stromal tumor and the relevance of the risk grade. Hum Pathol 2005;36:828-37.

36. Chen WT, Huang CJ, Wu MT, Yang SF, Su YC, Chai CY. Hypoxia-inducible factor-1alpha is associated with risk of aggressive behavior and tumor angiogenesis in gastrointestinal stromal tumor. Jpn J Clin Oncol 2005;35:207-13.

37. Kim TW, Lee H, Kang YK, Choe MS, Ryu MH, Chang HM, Kim JS, Yook JH, Kim BS, Lee JS. Prognostic significance of c-kit mutation in localized gastrointestinal stromal tumors. Clin Cancer Res 2004;10:3076-81.

38. Montgomery E, Abraham SC, Fisher C, Deasel MR, Amr SS, Sheikh SS, House M, Lilliemoe K, Choti M, Brock M, Ephron DT, Zahuruk M, et al. CD44 loss in gastric stromal tumors as a prognostic marker. Am J Surg Pathol 2004;28:168-77.

39. Kanat O, Adim S, Evrensel T, Yerci O, Ediz B, Kurt E, Demiray M, Gonullu G, Arslan M, Manavoglu O. Prognostic value of nm23 in gastrointestinal stromal tumors. Med Oncol 2004;21:53-8.

40. Takahashi R, Tanaka S, Hiyama T, Ito M, Kitadai Y, Sumii M, Haruma K, Chayama K. Hypoxia-inducible factor-1alpha expression and angiogenesis in gastrointestinal stromal tumor of the stomach. Oncol Rep 2003;10:797-802.

41. Toquet C, Le Neel JC, Guillou L, Renaudin K, Hamy A, Heymann MF, Simon-Valla S, Le Borgne J, Maugard C, Fiche M. Elevated (> or = 10%) MIB-1 proliferative index correlates with poor outcome in gastric stromal tumor patients: a study of 35 cases. Dig Dis Sci 2002;47:2247-53.
