# Supplementary figures and images for: Gene Expression Network Analysis of ETV1 Reveals KCTD10 as a Novel Prognostic Biomarker in Gastrointestinal Stromal Tumor
Source: PLoS One. 2013 Aug 19;8(8):e73896. doi: 10.1371/journal.pone.0073896 (PMC3747077; doi:10.1371/journal.pone.0073896)

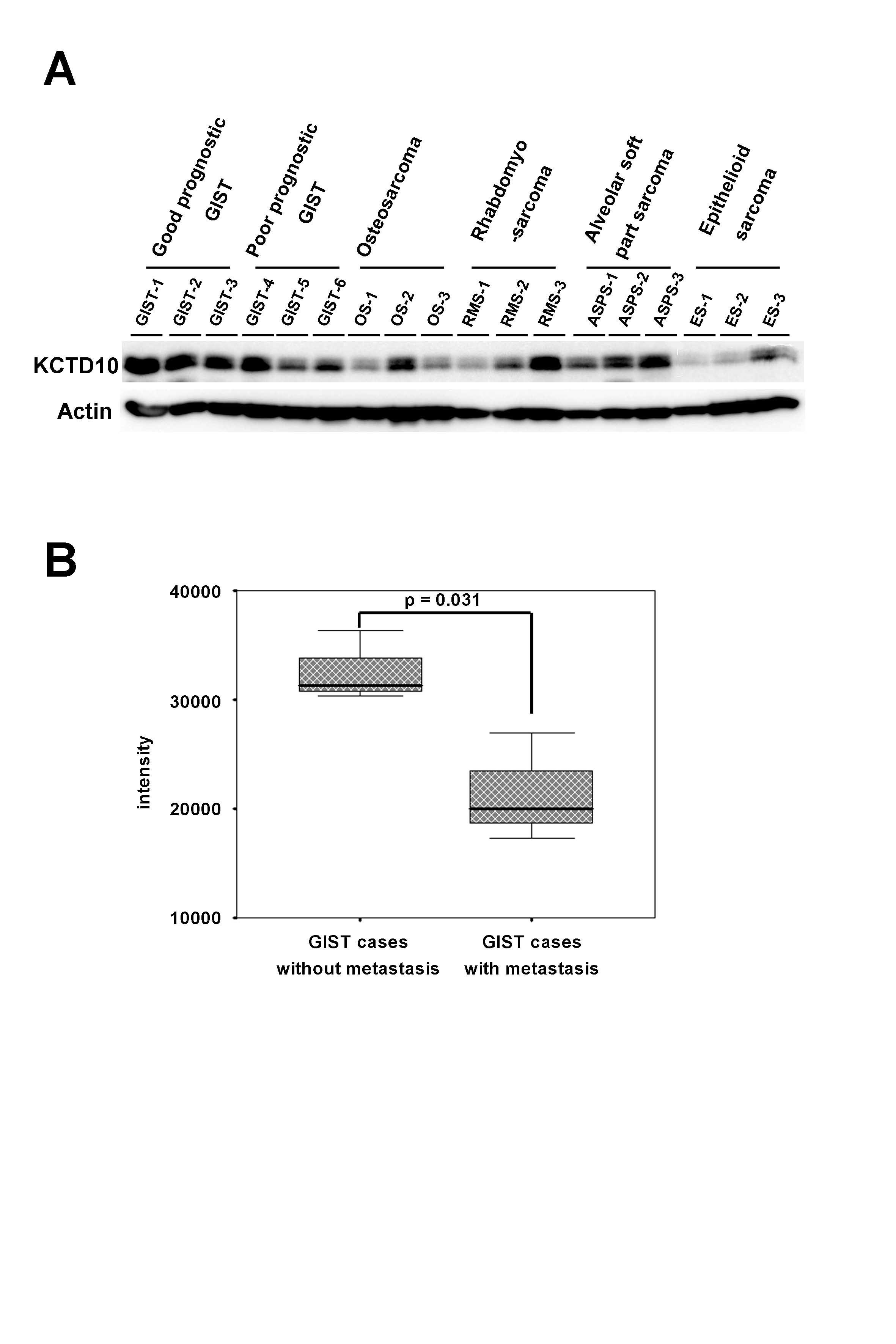

Supplement: Figure S1 — KCTD10 expression in GIST tissues evaluated by Western blotting (A). Expression of KCTD10 was broadly observed in sarcoma cases. Statistically significant differences in the expression level were observed between the patient groups with different prognosis (B). (JPG) [file pone.0073896.s002.jpg]
